# Genome-Wide Identification of Splicing Quantitative Trait Loci (sQTLs) in Diverse Ecotypes of *Arabidopsis thaliana*

**DOI:** 10.3389/fpls.2019.01160

**Published:** 2019-10-03

**Authors:** Waqas Khokhar, Musa A. Hassan, Anireddy S. N. Reddy, Saurabh Chaudhary, Ibtissam Jabre, Lee J. Byrne, Naeem H. Syed

**Affiliations:** ^1^School of Human and Life Sciences, Canterbury Christ Church University, Canterbury, United Kingdom; ^2^Division of Infection and Immunity, The Roslin Institute, University of Edinburgh, Edinburgh, United Kingdom; ^3^Centre for Tropical Livestock Genetics and Health, University of Edinburgh, Edinburgh, United Kingdom; ^4^Department of Biology and Program in Cell and Molecular Biology, Colorado State University, Fort Collins, CO, United States

**Keywords:** splicing quantitative trait loci (sQTL), *Arabidopsis thaliana*, alternative splicing, isoform usage, GWAS, adaptation

## Abstract

Alternative splicing (AS) of pre-mRNAs contributes to transcriptome diversity and enables plants to generate different protein isoforms from a single gene and/or fine-tune gene expression during different development stages and environmental changes. Although AS is pervasive, the genetic basis for differential isoform usage in plants is still emerging. In this study, we performed genome-wide analysis in 666 geographically distributed diverse ecotypes of *Arabidopsis thaliana* to identify genomic regions [splicing quantitative trait loci (sQTLs)] that may regulate differential AS. These ecotypes belong to different microclimatic conditions and are part of the relict and non-relict populations. Although sQTLs were spread across the genome, we observed enrichment for *trans*-sQTL (*trans*-sQTLs hotspots) on chromosome one. Furthermore, we identified several sQTL (911) that co-localized with trait-linked single nucleotide polymorphisms (SNP) identified in the Arabidopsis genome-wide association studies (AraGWAS). Many sQTLs were enriched among circadian clock, flowering, and stress-responsive genes, suggesting a role for differential isoform usage in regulating these important processes in diverse ecotypes of Arabidopsis. In conclusion, the current study provides a deep insight into SNPs affecting isoform ratios/genes and facilitates a better mechanistic understanding of trait-associated SNPs in GWAS studies. To the best of our knowledge, this is the first report of sQTL analysis in a large set of Arabidopsis ecotypes and can be used as a reference to perform sQTL analysis in the Brassicaceae family. Since whole genome and transcriptome datasets are available for these diverse ecotypes, it could serve as a powerful resource for the biological interpretation of trait-associated loci, splice isoform ratios, and their phenotypic consequences to help produce more resilient and high yield crop varieties.

## Introduction

Plants have evolved diverse and efficient genetic and physiological strategies to cope with environmental fluctuations. For an appropriate response, plants employ different regulatory mechanisms that can modulate genomic architecture and transcriptome composition to generate phenotypic diversity, allowing them to engender appropriate responses and occupy diverse niches (Frachon et al., 2018). The majority of protein-coding genes (∼90%) in plants contain introns, which must be removed by a process called pre-mRNA splicing to produce mature messenger RNAs (mRNAs) for translation. Due to differential exon and splice site usage, ∼70% of plant genes can be alternatively spliced (AS) to generate few to thousands of structurally and functionally different mRNA isoforms with different fates (Filichkin et al., 2010; Marquez et al., 2012; Shen et al., 2014; Klepikova et al., 2016; Zhang et al., 2017). Interestingly, the majority of splicing regulator genes in plants are subject to extensive AS and change the profile of their splicing patterns in response to various environmental stresses (Palusa et al., 2007; Zhang et al., 2013; Calixto et al., 2018). AS events in some plant-specific SR genes are highly conserved from single-cell green alga *Chlamydomonas* reinhardtii or moss *Physcomitrella* patens to Arabidopsis, suggesting the importance of their regulation in plants (Iida and Go, 2006; Kalyna et al., 2006). Plants employ AS to fine-tune their physiology and metabolism to maintain a balance between carbon fixation and resource allocation under normal as well as biotic and/or abiotic stress conditions such as pathogen infection, temperature, salt, drought, wounding, and light (Feng et al., 2015; Huang et al., 2017; Calixto et al., 2018; Filichkin et al., 2018; Hartmann et al., 2018; Liu et al., 2018; Seaton et al., 2018). For example, short read (Illumina) and long read (Iso-seq) RNA-Seq data from poplar leaf, root, and stem xylem tissues under drought, salt, and temperature fluctuations revealed changes in AS profiles that modulate plant transcriptome under abiotic stresses (Filichkin et al., 2018). Moreover, intron retention (IR) was found to be the predominant and variable type of AS across all treatments and tissue types (Filichkin et al., 2018). In wheat, global profiling of the AS landscape responsive to drought, heat, and their combination showed significant AS variation (Liu et al., 2018). Recent studies have shown that salt stress, high temperature, disease, and cold-stress alter AS patterns in Arabidopsis, grape, rice, and soybean (Liu et al., 2013; Feng et al., 2015; Filichkin et al., 2015; Lee et al., 2016; Jiang et al., 2017; Calixto et al., 2018). Similarly, AS plays a key role in biotic stress responses; for example, data from soybean show that PsAvr3c, a *Phytophthora* sojae pathogen effector, can manipulate host spliceosomal machinery to shift splicing profiles and overcome the host immune system (Huang et al., 2017). However, it is not clear to what extent sequence variation influences splicing variation and whether chromatin environment also contributes towards it, in a condition- and stress-dependent manner (Syed et al., 2012; Pajoro et al., 2017; Jabre et al., 2019).

Genome and transcriptome sequencing in multiple accessions of Arabidopsis revealed that genetic variations influence the expression and splicing of several genes, including stress-responsive genes (Gan et al., 2011). For instance, a strong association between genetic variations and spliced isoform accumulation was observed in sunflower (Smith et al., 2018). Interestingly, a significant proportion of splicing variation was associated with variants that harbor *trans*-QTLs, of which the majority were associated with spliceosomal proteins (Smith et al., 2018). Further examination of splicing variation in the wild and cultivated sunflowers revealed that higher frequency of AS was triggered during the domestication process. Some of the genetic variations can also influence important life-history traits like flowering and may have an influence on the geographical distribution of different accessions. For example, insertion polymorphisms in the first intron of the *FLOWERING LOCUS M* (*FLM*) gene influence AS and accelerate flowering in a temperature-dependent manner in many accessions of Arabidopsis (Lutz et al., 2015). Taken together, structural variation in the *FLM* gene can change ratios of different splice variants and influence a highly adaptive trait-like flowering in Arabidopsis (Lutz et al., 2015).

Insight into trait-associated genetic variants and their distribution pattern can delineate the mechanisms of genome regulation (Alonso-Blanco et al., 2016). Since AS can increase transcriptome/proteome complexity, the genetic underpinnings of natural sequence variations and AS are strongly associated with each other. Genetic variants, such as single nucleotide polymorphisms (SNPs), can substantially regulate the expression of transcript isoforms by modulating splice sites, which can impact phenotypic diversity and susceptibility to diseases in humans (Singh and Cooper, 2012; Takata et al., 2017). Recent studies showed that 22% of SNPs that are associated with different human diseases affect splicing (Qu et al., 2017; Park et al., 2018). The advances in RNA-Seq and genotyping have augmented the opportunities to monitor genetic variants and quantify transcriptomic features that allow us to understand the genetic landscapes of AS (Smith et al., 2018). Recent studies in animals and plants have elucidated the association of genetic variants with trait-associated loci at a population level (Monlong et al., 2014; Chen et al., 2018). In Arabidopsis, many studies have identified expression quantitative trait loci (eQTL) to explain trait-associated loci (Zhang et al., 2011). However, there have been very few studies on the genome-wide investigation of genetic variants affecting splicing patterns termed as splicing quantitative trait loci (sQTLs) in Arabidopsis (Yoo et al., 2016). To illuminate the role of genetic variations on AS in a large collection of highly diverse Arabidopsis lines, we sought to map sQTLs influencing AS. sQTLs spread across the genome can either act in *cis*- to disrupt the splicing of a proximal pre-mRNA by modulating splicing factors binding affinity to the pre-mRNA or in *trans* by regulating the splicing of distal pre-mRNA through altered expression of splicing regulators (Yoo et al., 2016; Qu et al., 2017).

We have used 666 diverse natural inbred Arabidopsis accessions including ‘relicts’ that occupied postglacial Eurasia first and were later invaded by ‘non-relicts’, which demographically spread along the east-west axis of Eurasia owing to its higher latitudinal regional diversity, human disturbance and climatic pressure (François et al., 2008; Alonso-Blanco et al., 2016). These accessions are of immense significance as they hold a huge amount of diversity and their expansion leaves traces of admixture in the north and south of the species range that facilitated colonization to new habitats (Lee et al., 2017). In order to illuminate the relationship between splicing variants, phenotypic diversity and geographical distribution of these lines, we have performed sQTL analysis to reveal the functional impact of genetic variations on AS and adaptive consequences (Han et al., 2017). This analysis will provide a solid platform in the form of a useful and well-enriched dataset for sQTL in Arabidopsis to develop more resilient plant species in the face of climatic challenges to crop production.

## Materials and Methods

### Genotype Datasets and Quality Control

High-quality genetic variant (SNPs) data for 1,135 globally distributed natural inbred lines of Arabidopsis representing relicts (accessions that hold ancestral habitats) and non-relicts (accessions range from native Eurasia to recently colonized North America) were downloaded in variant calling format (vcf) from the 1001 Genomes Data Centre (https://1001genomes.org/data/GMI-MPI/releases/v3.1/) (Alonso-Blanco et al., 2016). The genetic variants were pre-processed using stringent filtering criteria as follows: (i) having at least two genotypes across accessions, and (ii) each genotype has at least five occurrences across all accessions (Yang et al., 2017). Genotypes that occurred in less than five samples were converted to NA values to avoid their consideration in linkage analysis. The pre-processed high-quality genetic variant data was then used as input in mapping sQTLs (**Supplementary Figure 1**).

### RNA-Sequencing Analysis

Single-ended 100 bp long RNA sequencing (RNA-Seq) reads, generated by Illumina HiSeq 2500 platform, for 727 ecotypes of Arabidopsis (without biological replicates) were downloaded from GEO dataset under accession number GSE80744 and SRA study SRP074107 (Kawakatsu et al., 2016). Read quality assessment was performed using FastQC and reads with Phred score < 20 were removed *via* Trim Galore version 0.5.0 (Andrews, 2010; Krueger, 2015). Transcript abundance level in terms of transcripts per million (TPM), was estimated for 82,910 isoforms in 34,212 genes using Arabidopsis reference transcript dataset (AtRTD2) and Salmon (Zhang et al., 2015; Patro et al., 2017) (**Supplementary Dataset 1**). The filtered RNA-seq reads were also mapped to the TAIR10 genome assembly using the STAR aligner version 2.7.0e with modified parameters (**Supplementary Dataset 1**) (Dobin et al., 2013). Transcripts were assembled and assemblies were merged using StringTie (Pertea et al., 2015) (**Supplementary Dataset 4**). The merged assembly was then used as a reference gene model to perform the second assembly to generate expression dataset for 124,422 expressed transcripts using StringTie (Pertea et al., 2015). The TPM values for both (transcriptome- and genome-based) expression datasets were then used to compute splicing ratios of each isoform for all genes. Genes with less than two isoforms or splicing variability <0.01 were filtered out using the core functionality of ulfasQTL method (Yang et al., 2017).

### Identification of sQTLs

The genotype data from 1,135 and RNA-seq data from 727 Arabidopsis accessions were initially processed to filter 666 accessions that have both genotype and transcriptomic data (**Supplementary Table 1**; **Supplementary Figure 1**). The processed genetic variants and expression datasets were used to perform a genome-wide scan for sQTLs using the ulfasQTL package (v 0.1) – a composite sQTL analysis package that takes expression and genotype dataset to test splicing QTLs at genome-wide scale (Yang et al., 2017). It uses the core functionality of sQTLseekeR approach, which is a multivariate model and calculates splicing ratios variability of a gene across samples using a distance-based approach. It estimates intra- and inter- genotype splicing variability using a non-parametric analog to the ANOVA (Monlong et al., 2014). ulfasQTL identifies and outputs a list of significant sQTLs (p-value ≤ 0.05) and their cognate genes across the genome. We used sQTL cognate genes to derive modes of AS events present in these genes using SUPPA version 2.2.1 (Alamancos et al., 2014; Alamancos et al., 2015), which provides an estimate of the inclusion level of AS events across all samples.

### Colocalization of sQTL With GWAS Hits

The list of trait-associated loci was downloaded from the Arabidopsis genome-wide association studies (AraGWAS) catalog, which is a manually curated and standardized database that holds GWAS results for 167 publicly available phenotypes of Arabidopsis (Togninalli et al., 2018). It contains around 222,000 SNP-trait associations (GWAS hits), of which 3,887 are highly significant (p-value < 10^−4^). The list of unique sQTLs was then matched with trait-associated loci (identical SNPs) to identify the significant association with important phenotypic traits associated sQTLs.

### Gene Enrichment Analysis

Functional enrichment analysis was performed on the parent genes with significant sQTL using Database for Annotation, Visualization and Integrated Discovery (DAVID version 6.8) with default parameters that work on the principle of Fisher exact test for statistical analysis (Huang et al., 2009). The gene ontology (GO) terms [biological process (BP), molecular function (MF), and cellular components (CC)] were identified to provide biological insights into the significant sQTLs using false discovery rate (FDR) ≤0.05.

### Functional Annotation of sQTL and Non-sQTL SNPs

Publicly available functionally annotated SNPs dataset for 666 Arabidopsis accessions was downloaded and overlapped with sQTL-SNPs to obtain functional annotation of sQTLs and termed the other unmatched SNPs as non-sQTL SNPs. The SNPs were further classified into exonic-SNPs (nonsense, start-loss, frameshift, splice site, missense, synonymous, splice region, 5-UTR, 3-UTR, and non-coding exon variants) and non-exonic SNPs (intron and intergenic variants).

## Results

### Majority of Splicing Events in *A. thaliana* Are Regulated as *Trans*

**Figure 1:**
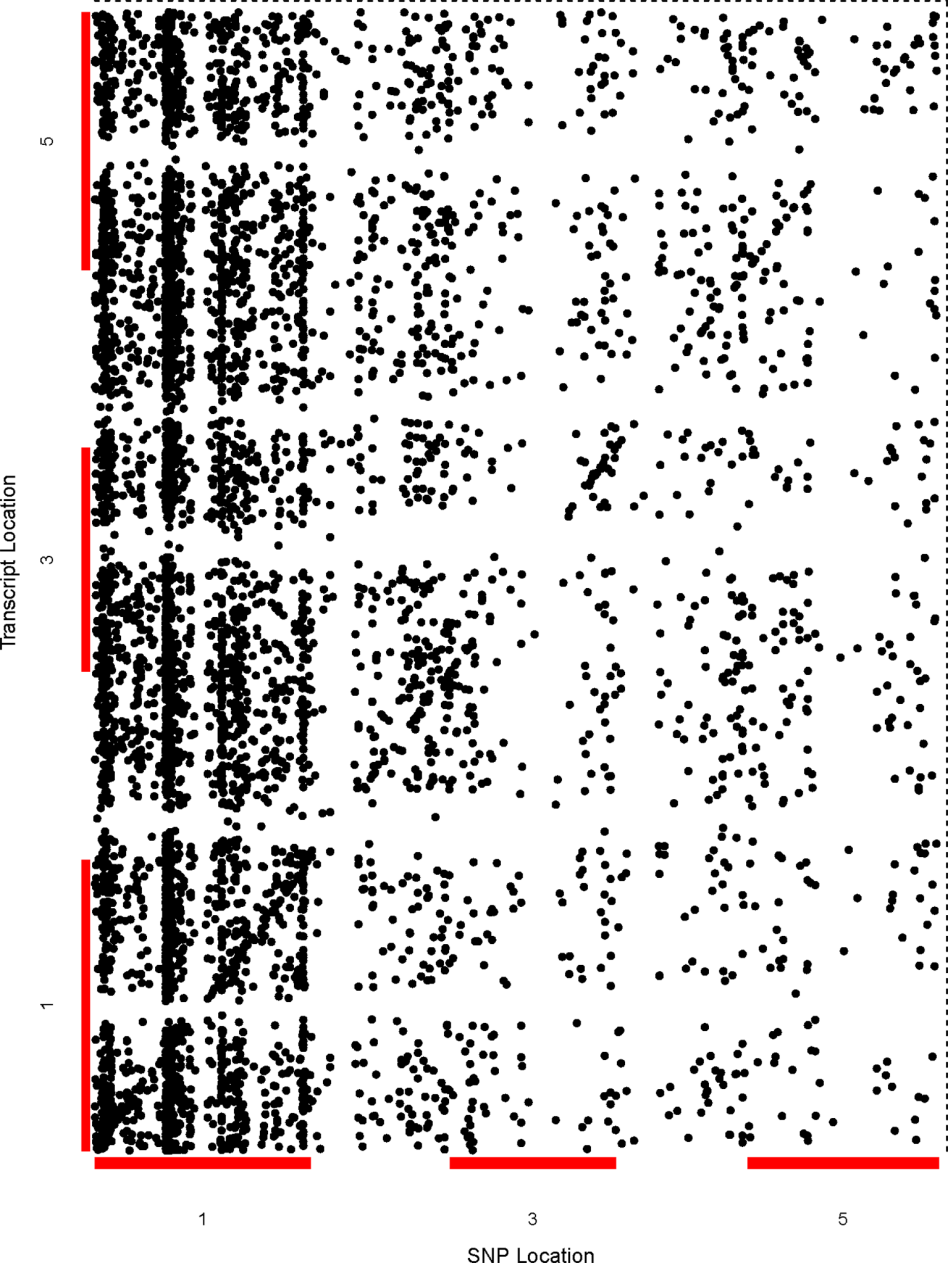
Genetic analysis shows the genome-wide distribution of sQTLs and enrichment of *trans*-sQTL on chromosome 1. A comprehensive two-dimensional genome-wide sQTL map showing the distribution of all significant signals for unique sQTLs. Each dot represents an association between a genetic variant location (x-axis) and cognate transcript location (y-axis). The points along the diagonal correspond to *cis*-associations (window 4 kb) with the vertical band representing *trans*- sQTLs.

**Figure 2:**
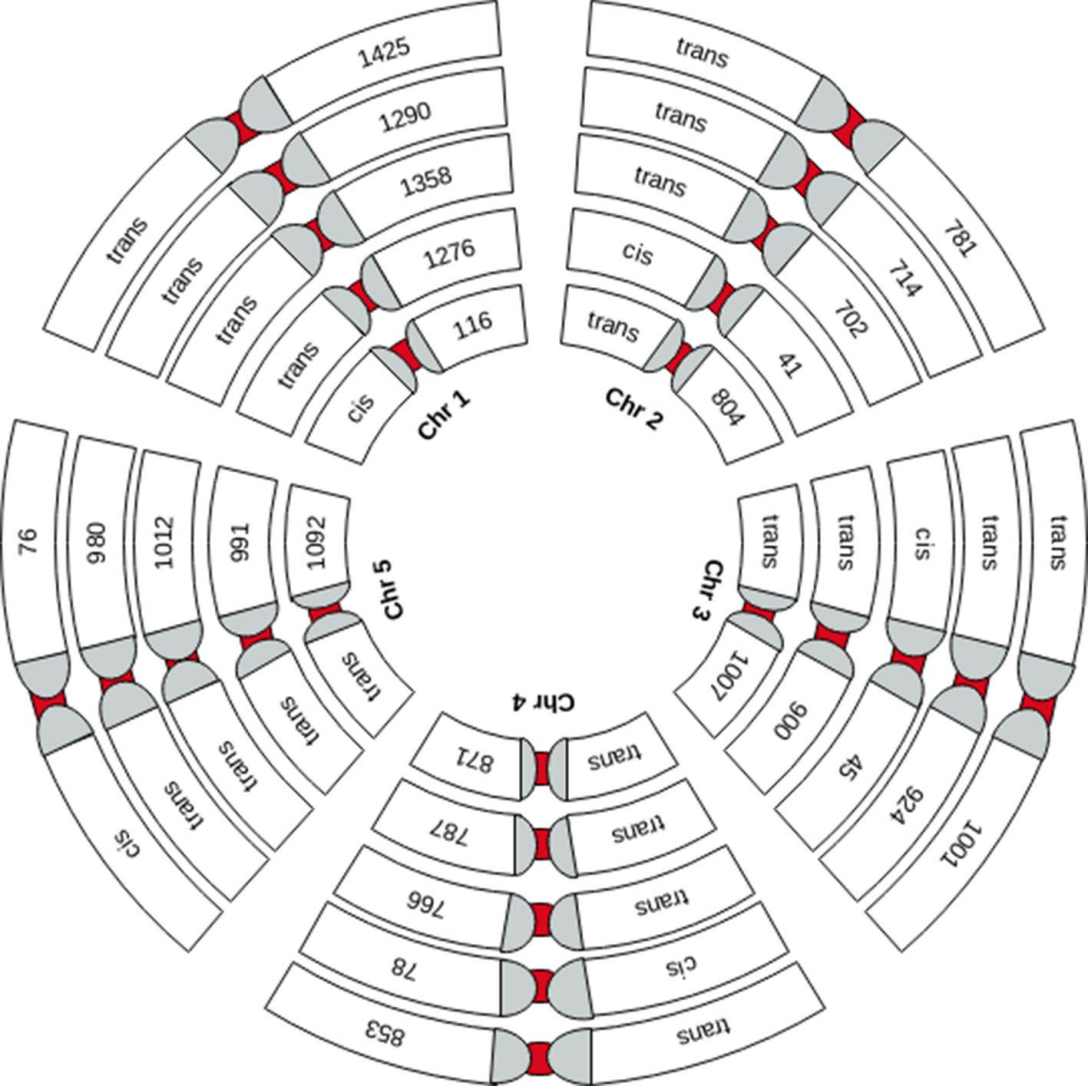
Graphical representation of the chromosome-wide distribution of sQTLs. Each group of five chromosomes (Chr1 to 5 is depicted by small to bigger circles, respectively) shows *cis*-sQTLs on one of the five chromosomes and *trans*-sQTLs on other chromosomes.

We performed genome-wide sQTL analysis using transcriptomic and genomic datasets and identified 6,406 and 6484 unique sQTLs that are associated with 6,129 and 7653 non-redundant genes, respectively (**Table 1**; **Supplementary Tables 2** and **3**). The comparison of sQTL analysis based on two expression datasets (AtRTD2 and genome assemblies) showed significant overlapping of sQTLs (6181) between two strategies (**Supplementary Figure 1**). The number of cognate genes increased for the genomic dataset, possibly due to the presence of novel genes/transcripts coming from known/novel genes. The number of transcripts present in the transcriptomic expression dataset is less, but these transcripts were experimentally validated in pilot studies so we used AtRTD2 transcriptomic expression dataset for further analysis. Although the sQTLs were randomly distributed across the genome, 1775 (∼28%) of sQTLs localized on chromosome one, whereas chromosome two had the lowest number (956; ∼15%) of SNPs linked to splicing patterns (**Table 1** and **Figure 1**). The higher distribution of sQTLs on chromosome one is probably due to its bigger size as compared with other chromosomes (Rhee et al., 2003). To get a better understanding of the influence of the genetic variants (SNPs) on splicing patterns, sQTLs that were within 4 kb from their cognate gene were designated as *cis*-sQTL and every other sQTL outside this window, including those on a different chromosome, as trans-sQTLs. Subsequently, 356 *cis*-sQTLs (5% of the total mapped sQTLs) that were associated with the splicing of 301 genes and an extensively high frequency (95%) of trans-sQTLs were identified (**Table 1** and **Figure 2**). Interestingly, an overrepresentation of *trans*-sQTLs (*trans*-sQTL hotspots) on chromosome one was observed, which indicates that the molecular factor(s) on this chromosome that may regulates the splicing of several transcripts are *trans*. The sQTLs were then mapped with a list of trait-associated SNPs available in GWAS catalog, and the exact match was found for 911 non-redundant SNPs associated with 757 genes (**Supplementary Table 4**). Among 911 sQTLs that overlapped with GWAS catalog SNPs, 61 are cis-sQTL and are associated with 48 different gene, while 850 are *trans*-sQTL and linked with 709 genes.

**Table 1:** Genome-wide summary of sQTL mapped using 666 diverse ecotypes of *Arabidopsis thalian* *a*.

Chromosome	Total sQTLs	Cis-sQTL	Cis-sQTL linked genes	Trans-sQTL	Trans-sQTL linked genes
Chr1	1,775	116	93	5,177	1,741
Chr2	956	41	35	4,688	1,111
Chr3	1,266	45	46	4,773	1,083
Chr4	1,055	78	60	4,641	1,052
Chr5	1,354	76	76	5,112	1,537

**Figure 3:**
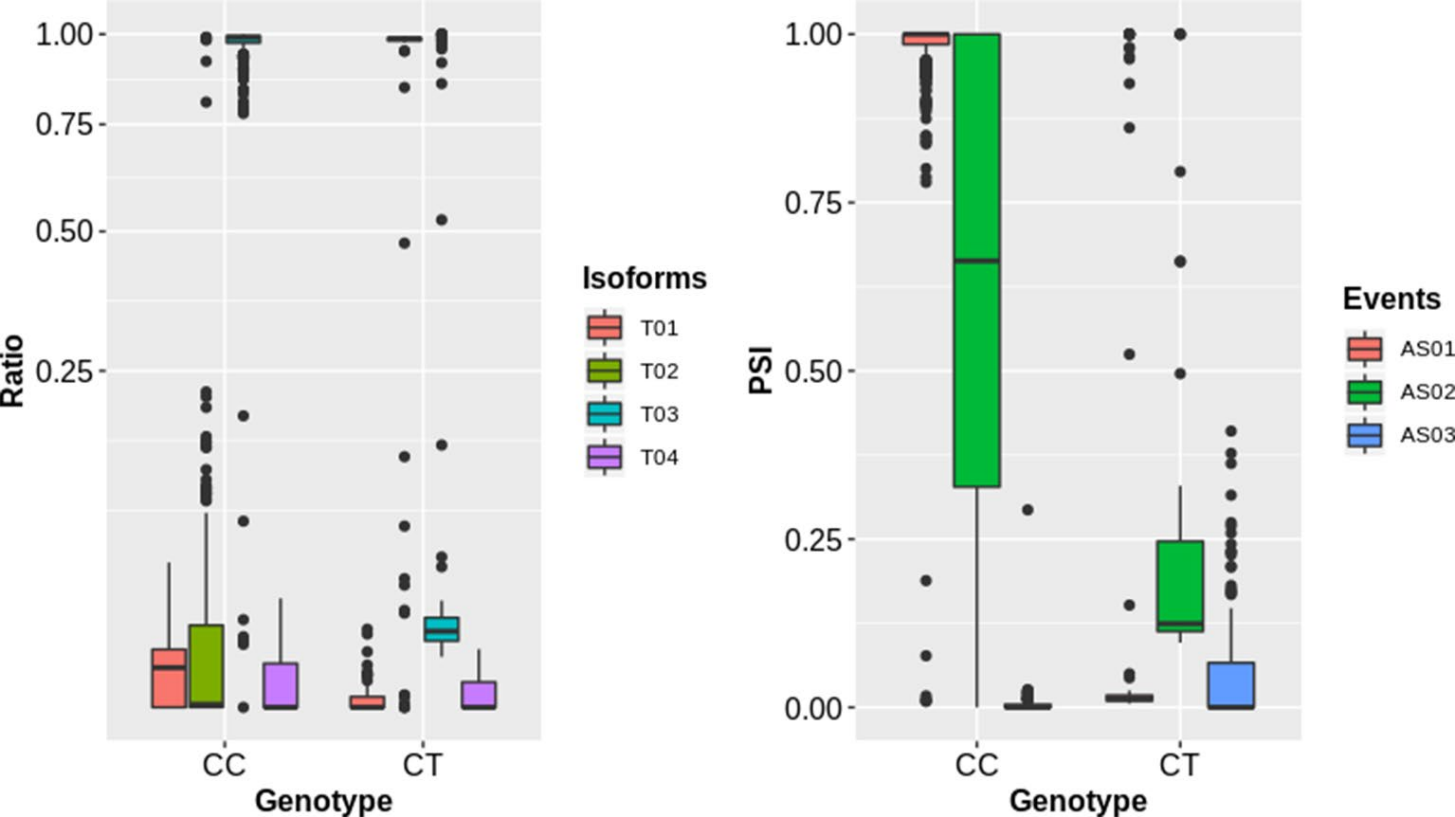
The impact of sQTLs (snp_1_1099063) on splicing isoform ratios and AS events of the gene (AT1G04170). The left panel shows the impact of change in genotype on splicing ratios of transcripts (T01: AT1G04170_c1, T02: AT1G04170_Jc4, T03: AT1G04170_P1, T04: AT1G04170_P2) with sharp change for T01 and T03. The right panel shows the splicing events (AS01, AT1G04170;A3:Chr1:1097120-1097399:1097120-1097405:+, AS02, AT1G04170;A3:Chr1:1097286-1097396:1097286-1097399:+, AS03, AT1G04170;A5:Chr1:1097286-1097399:1097120-1097399:+) with significant change for AS01 and AS02 event.

**Figure 4:**
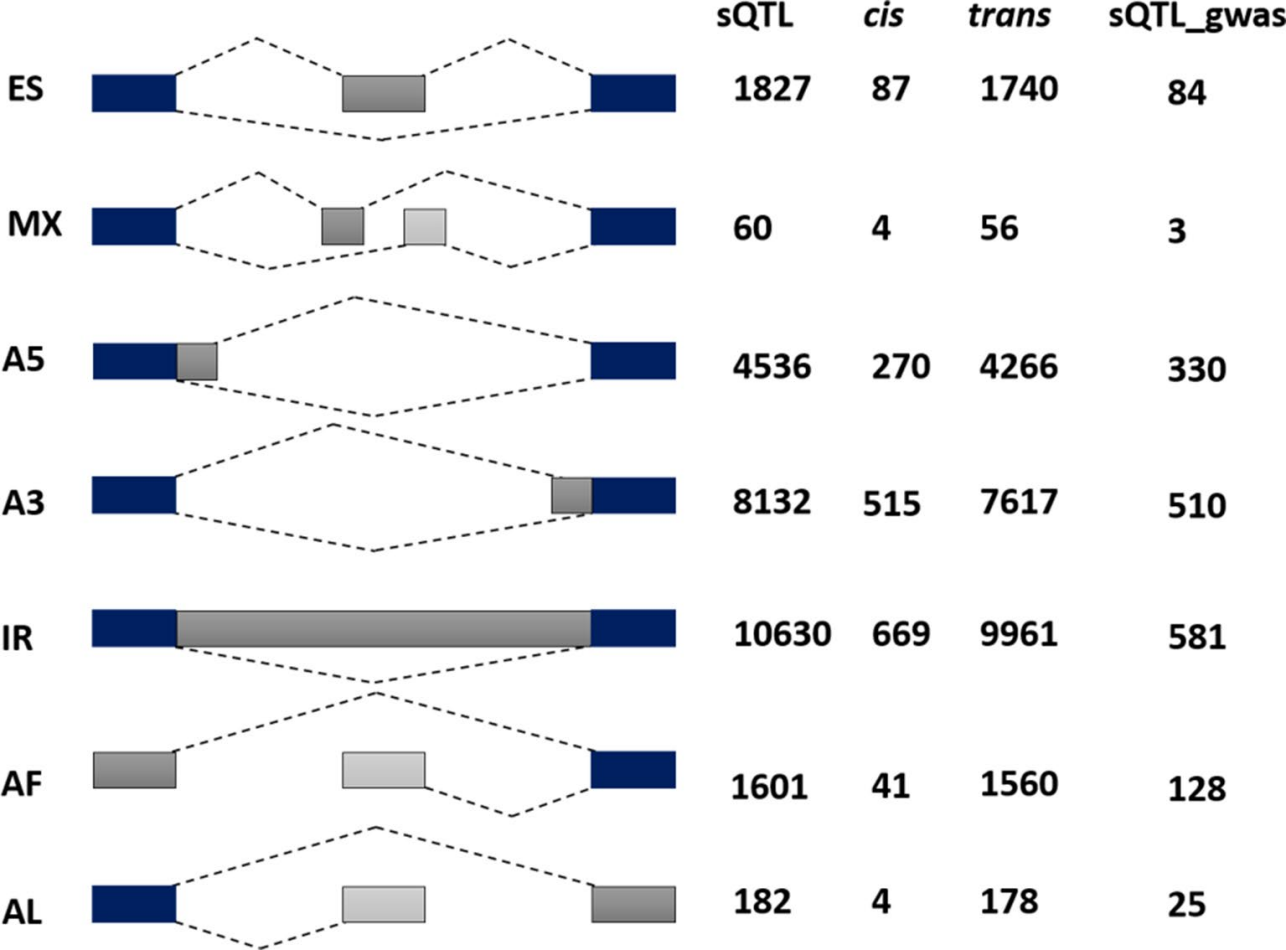
Alternative splicing (AS) categorization of 6129 significant sQTL cognate genes possessing 26968 AS events. Among these 1590 are associated with *cis* and 25378 with the *trans* category. The last column reflects AS pattern of 757 sQTL-GWAS cognate genes possessing 1661 AS events. Among AS categories (exon skipping (ES), mutually exclusive exons (MX), Alternative 5′/3′ splice-site (A5/A3), Intron retention (IR), Alternative First/Last Exons (AF/AL), IR are the most common, whereas mutually exclusive exons were the least frequent type of alternative splicing.

To complement the above analysis, we estimated the splicing ratios and AS categories of significant sQTL cognate genes (6129) containing 34,351 transcripts and 26968 AS events (**Supplementary Datasets 2** and **3**). The association of one of the top sQTL SNP that resides on chromosome 1 at position 1099063 with splicing ratios of *AT1G04170* (*EIF2 GAMM*A; *EUKARYOTIC TRANSLATION INITIATION FACTOR 2 GAMMA SUBUNIT*) showed that alteration in genotype from homozygote CC to heterozygote CT significantly modulates splicing ratios of the isoforms *AT1G04170_JC4 and AT1G04170_P1* (**Figure 3**). Further analysis based on Percent Spliced in (PSI) values highlights the impact of sQTL at AS event level that reflects a significant change in A3 events (**Figure 3**). The overall AS estimates of sQTL cognate genes shows that IR (10630) was the dominant and mutually exclusive exons (MX) (60) was the least frequent alternative splicing event in diverse accessions of A. *thaliana* (**Figure 4**). Although less than IR events, the number of alternative 3’ splice site (A3) events (8132) was significantly higher than the alternative 5’ splice site (A5) events (4536). The number of Alternative first exon (AF) (1827) was close to skipped exons (ES) (1601) but way higher than alternative last exon (AL) (182). The AS events (26968) were subset into *cis* (1590) and *trans* (25378) sQTL cognate gene categories and observed a similar pattern of splicing events. Furthermore, predominance for IR and lower frequency for MX was also observed when analyzed AS genes that emerge as a result of the overlapping of sQTL with GWAS. We also highlighted the subclass of IR events known as Exitrons that are present in coding exons and possess the exonic potential to significantly modulate proteome diversity (Marquez et al., 2015). To illuminate their contribution towards IR events, we extracted exitrons (exon-like introns) by overlapping a list of publicly available 2459 exitrons with 3798 genes possessing 10630 IR event and found 913 common genes (**Supplementary Table 5**). This AS analysis based on sQTL cognate genes revealed a significant role of IR events in shaping transcriptome diversity and may influence a plant adaptation to complex environmental conditions.

### sQTLs Are Enriched in Exonic Regions

**Figure 5:**
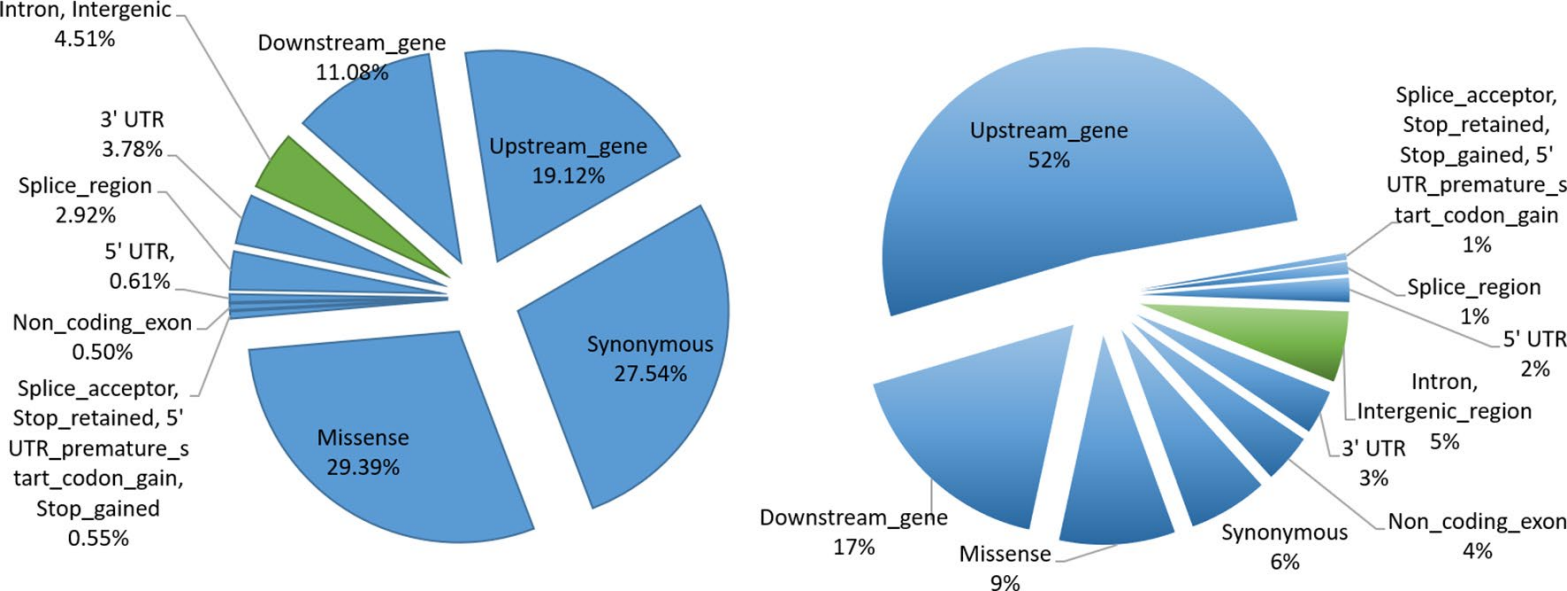
sQTL functional characterization. Pie charts indicating proportions of SNPs annotated with each functional category. SNPs in exonic and non-exonic regions are indicated by bluish and greenish colors, respectively. The left shows the functional categorization of sQTL SNPs, whereas the right panel depicts the functional categorization of non-sQTL SNPs.

In order to understand the biological significance of our results, we performed functional categorization of filtered SNPs (12,617,361) by classifying them into sQTLs (6,406) and non-sQTLs (12,610,954) SNPs. We were interested in better understanding their role in genome regulation, so we looked at the genomic distribution of sQTL/non-sQTL SNPs and characterized them as exonic SNPs and non-exonic SNPs. The sQTLs were enriched in exonic regions, which show their immense potential to modulate genomic architecture to generate phenotypic diversity. Among exonic variants, missense gene variants showed the highest frequency (1,883) followed by synonymous (1,764) and upstream gene (1,225) variant categories (**Figure 5**). Among non-exonic regions, sQTLs that reside in the intronic regions were significantly higher (282) than intergenic regions (7). Although the non-sQTLs were also enriched in exonic regions, yet within exonic regions, they painted a different picture as compared to sQTLs as they showed a higher number of upstream gene variant regions, compared with the rest of exonic non-sQTLs. However, the proportion of both categories (intron, intergenic) of non-exonic non-sQTLs is almost similar. Analysis of the functional context of sQTLs provided by SnpEff revealed a high impact on splice acceptor and stop gained functional categories, although their occurrence in sQTLs is low. The summary of all exonic and non-exonic SNPs for both functional classes (sQTLs and non-sQTLs) showed around 95% SNPs are enriched in variants belong to exonic regions and the majority of them represents the *trans* category, while only 5% were found in intronic regions. Furthermore, we have analysed the distribution of *cis* and *trans*-sQTLs along with the exonic locations. Of the 356 *cis*-sQTLs, significantly more (342) reside in exonic locations (hypergeometric p-value ≤ 0.05). Similarly, significantly more (5,775 out of 6,050) *trans*-sQTLs localized in exonic regions (hypergeometric p-value ≤ 0.05).

### Biological Significance of sQTL Cognate Genes

**Figure 6:**
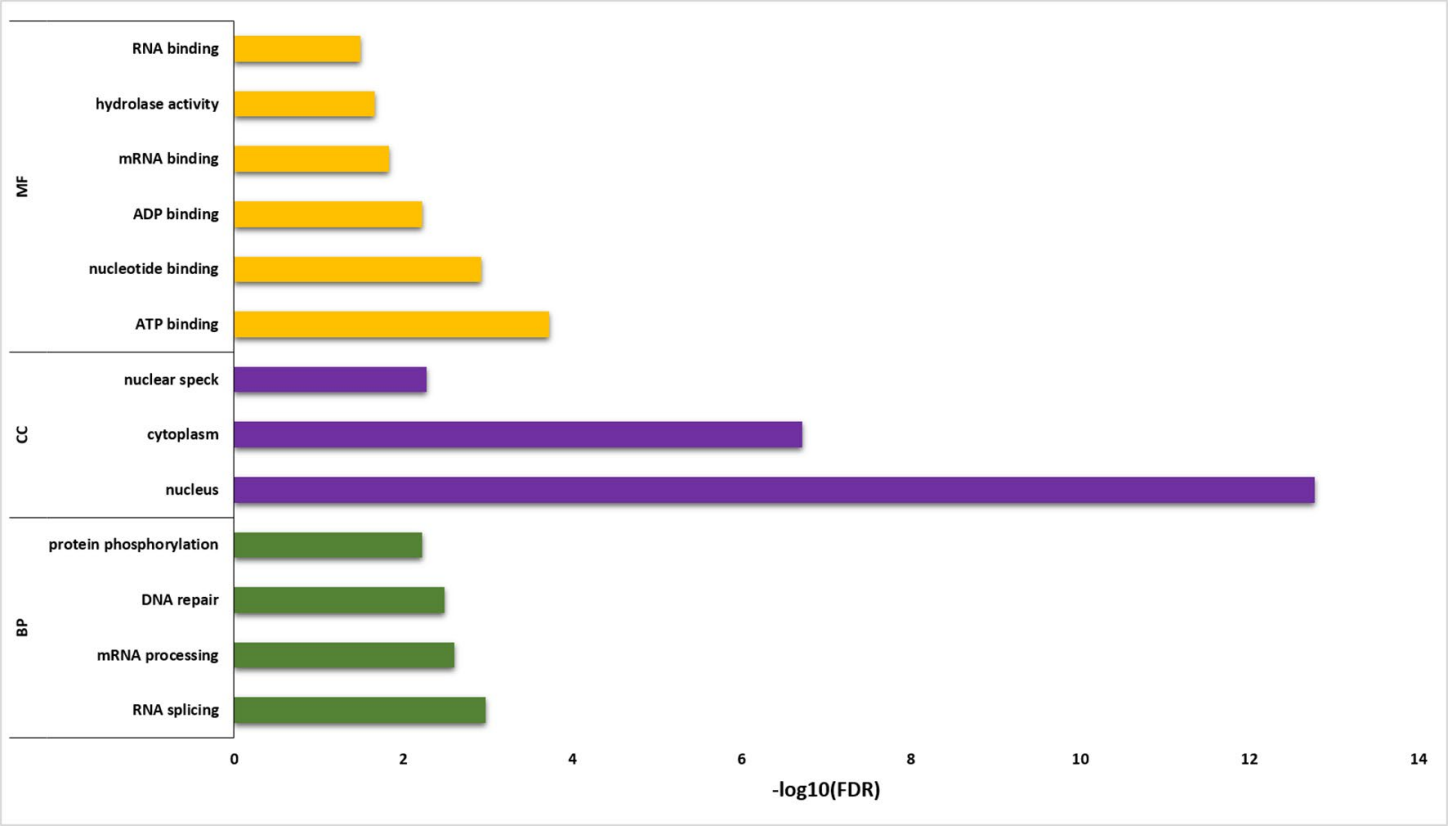
Gene enrichment analysis for sQTL-associated genes. Y-axis shows the gene enrichment categories, whereas X-axis illustrates –log10 (FDR). BP, CC, and MF represent a biological process, cellular component, and molecular function, respectively.

Functional annotation analysis of 6,129 sQTL cognate genes was performed using DAVID (Huang et al., 2009) (**Figure 6**; **Supplementary Table 6**). The statistically significant gene enrichment terms were filtered based on FDR ≤ 0.05 to illuminate the momentous role of sQTL cognate genes in diverse biological processes, cellular components, and molecular function. The involvement of sQTL cognate genes in complex biological processes like RNA splicing/processing shows its tremendous potential to modulate the transcriptomic architecture and ability of sQTLs to affect genes associated with DNA repair speculates its critical involvement in genome stability. Furthermore, sQTL association with post-translational modifications in the shape of protein phosphorylation implies their proteome regulatory role that can generate phenotypic diversity.

The presence of sQTL cognate genes in vital cellular components (nucleus, cytoplasm, nuclear speck) and its association with significant binding (RNA, mRNA, ATP, ADP) and catalysis (hydrolase activity) molecular functions highlights its involvement in integral cellular processing that can help in a plant adaptation to the microclimatic conditions.

### Genome Regulatory Role of sQTL

sQTL enrichment within annotated genome regulatory regions was analyzed as mapped sQTLs were spread across the genome and can be enriched among various genome regulatory elements. Moreover, transcription factors (TFs) are important genome regulators as they can mediate transcription by binding in the upstream region of their target genes (Jin et al., 2017). Therefore, the list of TFs from the Plant Transcription Factor Database (PlantTFDB) v4.0 (Jin et al., 2017) was downloaded and showed that sQTLs for 389 genes overlapped with 51 TF families.

Furthermore, the binding of regulatory proteins to *cis*-regulatory DNA elements (CREs) can orchestrate gene expression. DNAse I hypersensitive sites (DHSs) are significantly enriched in CREs that provide chromatin accessibility to regulatory proteins. The DHS sites and nucleosome positioning/occupancy for Arabidopsis were downloaded from PlantDHS database (Zhang et al., 2016) and revealed a significant overlap of sQTLs with CRE enriched regions. The leaf and flower tissue nucleosome positioning data of Arabidopsis was used to see the frequency of sQTLs that reside in nucleosome enriched regions and revealed that 462 sQTLs are flowering specific, 399 are leaf specific, and 4,962 are shared between both tissues. The list of 395 A. *thaliana* splicing-related genes from SRGD (splicing-related gene database) was downloaded to interrogate any overlap with non-redundant sQTL cognate genes and found 128 (7 *cis*- and 121 *trans*-associated) overlapping with splicing-related genes (**Supplementary Figure 2**; **Supplementary Table 7**). Among these splicing-related genes (128), the highest number was found on chromosome one (31) and least number on chromosome two (18), which also relates to chromosome size. The overlapped splicing-related genes (128) were associated with 2,397 sQTLs (∼37% of overall sQTLs), which show their tremendous potential to serve as significant genome regulatory elements.

### sQTLs Are Enriched Among Stress Responsive, Clock, and Flowering Genes

In order to understand the underlying dynamics of sQTLs with the spatial distribution of 666 accessions worldwide, we analyzed three highly significant gene functional categories (stress response, flowering, and circadian clock) among sQTL associated genes. The three categories are intimately associated with each other and confer adaptation to different climatic regions. Towards this goal, a list of 3150 Arabidopsis stress-responsive genes was downloaded from STIFDB2 database (Naika et al., 2013). In total, 742 stress-responsive genes associated with significant sQTLs were identified, highlighting the potential of AS in the plant stress response mechanism. Next, we downloaded a list of 346 flowering genes (306 flowering time and 46 flowering development genes) from FLOR-ID database (Bouché et al., 2016) and identified 122 genes that were associated with sQTLs. Similarly, out of 28 core clock genes, 16 were found to be associated with sQTLs (**Supplementary Figure 3A**).

Interestingly, we found six common genes (**Table 2**; **Supplementary Figure 3B**) between the three groups (circadian, flowering, and stress) that were associated with sQTLs. Besides core clock components like *circadian clock-associated 1* (*CCA1*), *late elongated hypocotyl* (*LHY*), *timing of cab expression 1* (*TOC1*), and pseudo response *regulator 7* (*PRR7*), we found *phytochrome interacting factor 5* (*PIF5*) and *b-box domain protein 19* (*BBX19*) genes, which are associated with light-sensing, flowering, and photomorphogenesis, respectively (Suárez-López et al., 2001; Hayama and Coupland, 2003; Somers et al., 2004; Wang et al., 2014; Greenham and McClung, 2015; Wang et al., 2015; Wang and Dehesh, 2015; Nasim et al., 2017; ). Since circadian clock and *PIFs (PIF 4/5* and others) influence the timing of flowering by modulating the expression of flowering-related genes, all accessions were divided on the basis of the timing of flowering at 10°C and 16°C (**Supplementary Figure 4**). Clock genes not only control global transcription patterns but their transcript abundance is also modulated by AS (James et al., 2012). Therefore, the mean expression of the aforementioned six genes revealed whether the expression of these genes is associated with the flowering time at 10°C and 16°C. Interestingly, expression of *LHY*, *CCA1*, and *PIF5* is significantly higher for plants growing at 16°C and is accompanied by the low expression of *TOC1* and *BBX19* (flowering repressor) among accessions that flower between 51 and 60 days. The relationship between the expression of these genes and flowering between 61 and 110 days is not straightforward; however, the expression of *PIF5* tremendously increased among accessions that flower late and is accompanied by a lower level of the expression of *BXX19*, which represses flowering by sequestering the *Flowering Time* (*FT*) gene (Wang et al., 2014). On the contrary, the expression of *PIF5* is generally higher among accessions flowering between 51 and 110 days at 16°C; however, late-flowering (131–160 days) accessions show a dramatic increase in the expression of *LHY*. Since most of the late flowering accessions are from Sweden, photoperiod-dependent flowering regulation *via LHY* is more pronounced (Park et al., 2016). Overall, these results indicate that clock, *PIF5*, and *BXX19* gene mediated flowering time is important for diverse accessions to occupy different geographical regions and many sQTLs mediate these responses.

**Table 2:** Association of six genes with sQTL-SNPs and GWAS-SNPs.

Genes ID	Gene name	Association with sQTL	Association with GWAS SNPs
AT5G02810	PRR7	344	18
AT3G59060	PIF5	44	10
AT1G01060	LHY	38	18
AT4G38960	BBX19	38	10
AT2G46830	CCA1	2	13
AT5G61380	TOC1	2	5

## Discussion

In this study, we analyzed population-scale transcriptomic and genotypic data of highly diverse 666 *A. thaliana* accessions to comprehensively identify the genomic regions regulating splicing (sQTLs). We used the AtRTD2 database as well as our own genomic assemblies to map sQTLs and found a significant overlap between the two approaches. Since there is significant overlap and the AtRTD2 database is highly validated and non-redundant, we suggest following the transcriptomic approach for sQTL mapping in Arabidopsis and other species where accurate datasets are available. Furthermore, using available transcriptomic datasets and Salmon based approaches are rapid and give reliable results without creating own transcriptome assemblies against the genome. The sQTLs based on the transcriptomic approach are spread genome-wide; however, their frequency varies across chromosomes. The chromosomal distribution relative to the genomic location of their cognate genes (*cis* or *trans*) showed that chromosome one harbors the highest number of *trans*-sQTL hotspots. The chromosomal distribution relative to the genomic location of their cognate genes (*cis* or *trans*) showed that chromosome one harbors the highest number of *trans*-sQTL hotspots. Among the top five associations, two of them reside on chromosome one and belongs to *cis* (snp_1_1099063; gene *AT1G04170*) and *trans* (snp_1_10688832; gene *AT1G30320*) categories; this strengthens our observation and reflects the immense potential of sQTLs to contribute towards genome complexity and proteome diversity. In order to gain more insight into the biological role and genetic control of splicing, we co-localized the sQTLs with Arabidopsis GWAS hits to illuminate their association with different trait-associated loci and their potential to modulate plants phenotypic variability (Yoo et al., 2016). Functional analysis of sQTLs showed that these genetic variants are significantly localized within exonic regions. Among the exonic regions variants, we discovered a high proportion of missense variants, which can modulate the structure and function of proteins. However, functional annotation painted a different picture by showing the moderate effect of missense variants but the high impact of stop gained and splice acceptor variants, which can modulate AS patterns and proteome diversity (Wachsman et al., 2017).

Although all classes of AS contributed to the sQTLs, intron retention (IR) was prevalent than any other AS type. This is consistent with the previous observations of IR as the most common class of AS and a well-established mechanism for regulating gene expression in plants (Syed et al., 2012; Reddy et al., 2013). However, how IR contribute and/or modulates proteome complexity and the extent to which IR sQTLs influence phenotypic variability remains obscure (Chaudhary et al., 2019). Although IR is associated with nonsense-mediated decay (NMD), this is by no means the only consequence as transcripts can still escape NMD *via* sequestration in the nucleus and some are preferentially recruited to ribosomes (Palusa and Reddy, 2010; Kalyna et al., 2012; Marquez et al., 2012). While our analysis showed that the majority of sQTLs are associated with IR events, most SNPs were localized within exonic regions. This is conceivable because only 356 *cis* sQTLs fall in this category and may have a subtle effect on IR in the *cis*-regulatory context. It is plausible that exonic variants cause changes in the pre-mRNA secondary structure, which impacts spliceosome recognition of exon–exon junctions (McManus and Graveley, 2011).

We interrogated the relationship between sQTL associated genes and annotated genome regulatory proteins and found a number of TF families linked to sQTLs. Although experimental validation is required to understand the regulation of TFs *via* AS, these results elaborate the potential of splicing as a mechanism for regulating gene expression (Nasim et al., 2017; Takata et al., 2017). Similarly, we also found a strong overlap between sQTLs, CREs, and nucleosome occupancy, especially among flower and leaf specific genes. Since nucleosome occupancy is much higher in exons, it helps to define intron–exon definition (Schwartz et al., 2009) to orchestrate an appropriate splicing response under variable environmental conditions. In addition, nucleosome occupancy has a strong influence on RNA polymerase II processing as its speed tends to be higher in regions with more open chromatin structure and Pol II speed regulates AS (Ullah et al., 2018; Godoy Herz et al., 2019). It is therefore not surprising that many sQTLs are enriched in the DHS site and have a strong association with regions with higher nucleosome occupancy among important life-history traits like leaf and flower. This data also indicates that many of the important traits like flowering are regulated *via* epigenetic means, and this is reminiscent of the downregulation of the *flowering* repressor *locus c* (*FLC*) that regulates flowering in a cold-dependent manner (Michaels and Amasino, 1999; Shindo et al., 2006).

Functional characterization of sQTLs among stress-responsive, circadian clock, and flowering genes revealed six common genes (*CCA1*, *LHY*, *TOC1*, *PRR7*, *PIF5*, and *BXX19*) that are shared in these categories (**Supplementary Figure 5**, **Supplementary Table 8**). Among these six genes, *PRR7 (AT5G02810)* showed high significance in the sQTL analysis by accruing second rank (**Supplementary Dataset 2**). *PRR7* was impacted by the trans-sQTL (*snp_5_626009*) present on chromosome 5 at position 626009, and this SNP modulated the splicing ratios and affected the AS patterns. We are aware that co-localisation of sQTLs in these categories would need further validation but we hope that it would provide an interesting starting point and a list of useful genes that may be regulated *via* alternative splicing. It is well known that the circadian clock plays a vital role in the normal functioning of plants and is intimately associated with carbon fixation during the day and starch mobilization to promote growth during the night (Dodd et al., 2005; Graf et al., 2010). Rhythmicity of clock components is not only associated with appropriate growth responses under variable and often stressful conditions but also promotes fitness and adaptive responses in plants (Dodd et al., 2005). Interestingly, down-regulation of *LHY* and *CCA1* around noon time among Arabidopsis hybrids and polyploids promotes heterosis *via* upregulation of chlorophyll, starch synthesis, and metabolism genes (Ni et al., 2009). Intriguingly, *1-aminocyclopropane-1-carboxylate synthase* (*ACS*, a rate-limiting enzyme in ethylene synthesis) is also downregulated by *CCA1* and phytochrome-*interacting factor 5* (*PIF5*) during the day and night, respectively, to promote heterosis in Arabidopsis (Song et al., 2018). Further, *LHY* and *CCA1* have been implicated in cold temperature acclimation responses and may also be important under higher temperature and stressful temperature conditions. *CCA1 and LHY* are partially redundant but show different expression and splicing patterns under cold conditions (Calixto et al., 2018). Similarly, *PRR7* has been associated with temperature responses in Arabidopsis and also shows different splicing patterns under normal and cold conditions (James et al., 2012). Recent experimental and modeling data suggest that *TOC1*, in concert with *ABA* levels, plays the role of an environmental sensor that coordinates the pace of the central oscillator to affect downstream processes (Pokhilko et al., 2013). Mechanistic details of *TOC1* mediated drought responses were revealed by analyzing the relationship between *TOC1* and an *ABA*-related gene (*ABAR/CHLH/GUN5*) (Legnaioli et al., 2009; Castells et al., 2010). Under drought conditions and elevated *ABA* levels, *TOC1* binds the *ABAR* promoter and modulates its circadian expression, resulting in clock-dependent gating of *ABA* function and drought tolerance (Legnaioli et al., 2009). Recent findings revealed that *LHY* modulates the expression of many genes involved in *ABA* signaling pathway to fine-tune plant performance under drought and osmotic stress conditions. However, *LHY* also maintained seed germination and plant growth *via* alleviation of the inhibitory effect of *ABA* (Adams et al., 2018).

The timing of flowering is crucial for the survival and adaptation of diverse Arabidopsis accessions in different geographical regions of the world. It is well known that the timing of flowering has a huge impact on seed set, grain filling, maturation, and overall productivity. Intriguingly, plants can also speed up their flowering in response to various environmental fluctuations and stresses, presumably as a survival strategy to produce seeds as quickly as possible (Wang and Dehesh, 2015). It is not surprising that one of the common genes between clock, flowering, and stress-related genes is *BBX19*. This gene has been shown to work as a circadian clock output and downregulates constans (*CO*) and precisely times the expression of *flowering* locus T (*FT*) in a day-length dependent manner to orchestrate appropriate flowering time (Wang et al., 2015). Interestingly, E3 ubiquitin ligase *constitutive photomorphogenic 1* (*COP1*), *early flowering 3* (*ELF3*), *phytochrome-interacting factor 4* (*PIF4*), and *PIF5* also influence *BBX19* function to mediate photomorphogenic responses in Arabidopsis (Wang et al., 2015). Furthermore, expression of *BBX19* is significantly reduced as a result of high levels of *methylerythritol cyclodiphosphate* (*MEcPP*), which is a plastidial isoprenoid intermediate that also functions as a stress-response retrograde signal to orchestrate appropriate transcriptional response (Xiao et al., 2012; Wang and Dehesh, 2015). Therefore, the *BBX19* gene provides the role of a flowering checkpoint that links a stress-specific retrograde signal (*MEcPP*) *via* sequestering the active *CO* gene, which is essential for *FT* transcription to promote flowering (Wang and Dehesh, 2015). Higher expression of *BBX19* delays flowering; however, its expression is positively correlated with *PIF5* expression to promote hypocotyl growth. Taken together, *BBX19* plays a dual role to modulate plant growth and stress-responsive flowering (Wang et al., 2015). Therefore, it is not surprising that many sQTL SNPs are associated with these genes and may be important for defining phenotypes and underlying genotypes among geographically diverse lines of Arabidopsis. Since *BBX19* controls flowering in a clock-regulated and stress-dependent manner, we propose that expression and sQTL patterns of *BBX19* may have a bearing on the flowering patterns of 666 accessions from different geographical regions of the world and play a role in adaptive responses. Recent evidence also shows that SNPs are present at almost every 200 bp in different ecotypes of Arabidopsis and can alter genomic architecture to affect splicing efficiency, gene expression pattern, and phenotypic diversity (Gan et al., 2011). We envisage that the presence of widespread variation in diverse ecotypes of Arabidopsis and our sQTL analysis would provide a solid platform for sQTL discovery and their influence on phenotypic traits in the future.

## Data Availability Statement

Publicly available datasets were analyzed in this study. Genotype data can be found here: https://1001genomes.org/data/GMI-MPI/releases/v3.1/ and RNA-Seq data can be found here: http://signal.salk.edu/1001.php

## Author Contributions

NS and WK conceived the study, WK performed most of the analysis, and MH, AR, SC, and IJ contributed to it. All authors contributed toward preparing the manuscript. WK and MH contributed equally.

## Conflict of Interest

The authors declare that the research was conducted in the absence of any commercial or financial relationships that could be construed as a potential conflict of interest.

## Supplementary Material

Supplementary Figure 1 Complementarity of the genome and transcriptome-based approaches for sQTL analysis.

Supplementary Figure 2 Summary of sQTL cognate genes overlapping with splicing related genes. The x-axis shows the type of splicing related genes and Y-axis exhibits the number of input and matched splicing related genes.

Supplementary Figure 3 Functional characterization of genes associated with sQTL. **(A)** sQTL cognate genes were highly enriched in different core functional categories (e.g. stress response, flowering). **(B)** Six genes were shared between different functional categories.

Supplementary Figure 4 Phenotypic association of six genes with flowering time. The x-axis shows days of flowering at 10 °C **(A)** and 16 °C **(B)** and Y-axis show the average/relative gene expression value for six genes across a diverse set of 666 A. thaliana accessions.

Supplementary Figure 5 The impact of sQTLs on splicing isoform ratios and AS events of six genes. The left panel shows the impact of change in genotype on splicing ratios of transcripts and the right panel shows the splicing events. For a detailed description of all isoforms and transcripts (see **Supplementary Table 8**).

Supplementary Dataset 1 RNA-Sequencing Analysis using genome as a reference

Supplementary Dataset 2 (Splicing ratios of sQTL cognate Genes): https://figshare.com/s/4dbd07a41f0fd4c6988c

Supplementary Dataset 3 (Percent Spliced In (PSI) of sQTL cognate Genes): https://figshare.com/s/89a7f23e21d7de9643c3

Supplemental Dataset 4 (Stringtie merged assembly): https://figshare.com/s/fd1a95af9e2b3025b8ff
