# Supplementary figures and images for: Genome-Wide Identification of Splicing Quantitative Trait Loci (sQTLs) in Diverse Ecotypes of Arabidopsis thaliana
Source: Front Plant Sci. 2019 Oct 3;10:1160. doi: 10.3389/fpls.2019.01160 (PMC6785726; doi:10.3389/fpls.2019.01160)

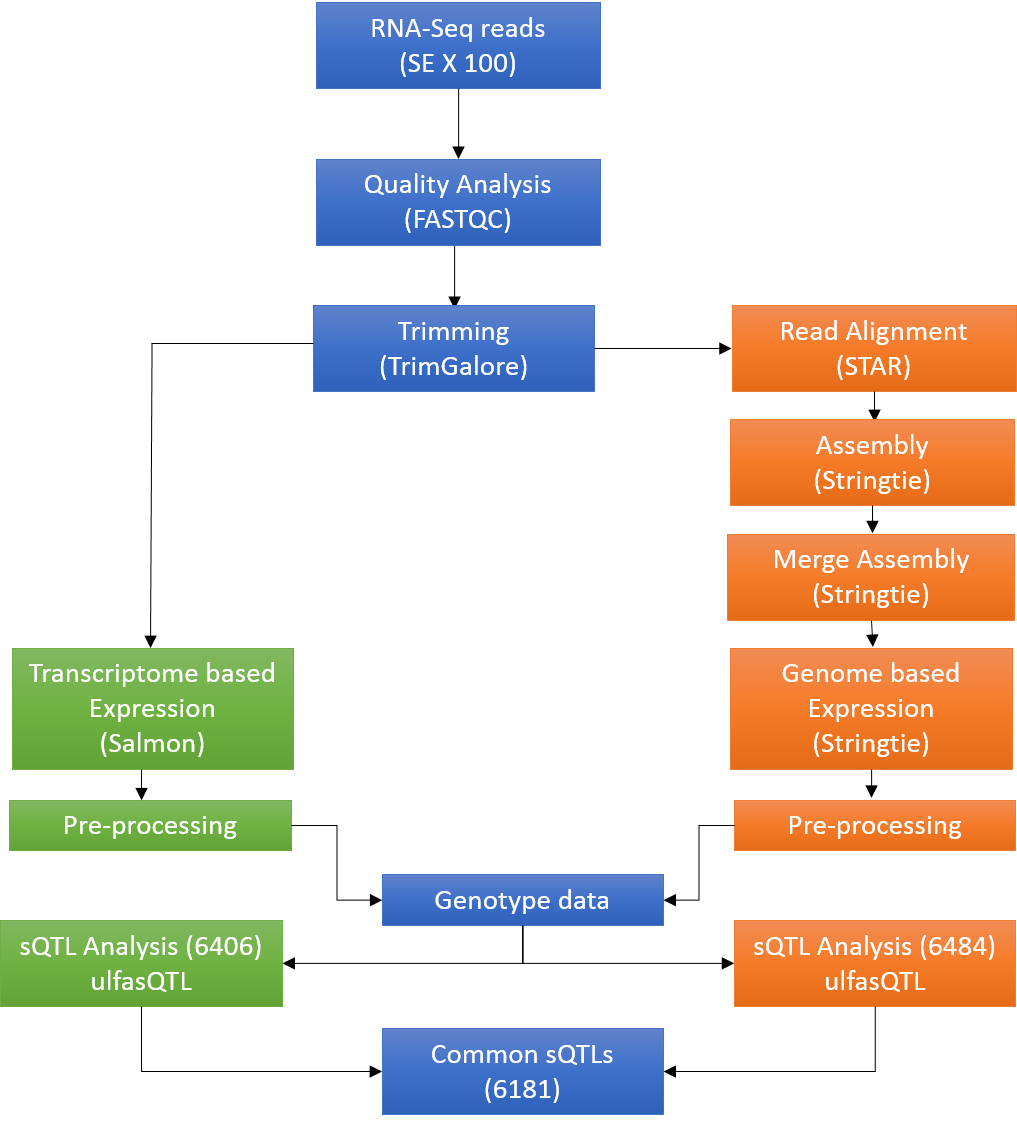

Supplement: Supplementary Figure 1 — Complementarity of the genome and transcriptome-based approaches for sQTL analysis. [file Image_1.tif]

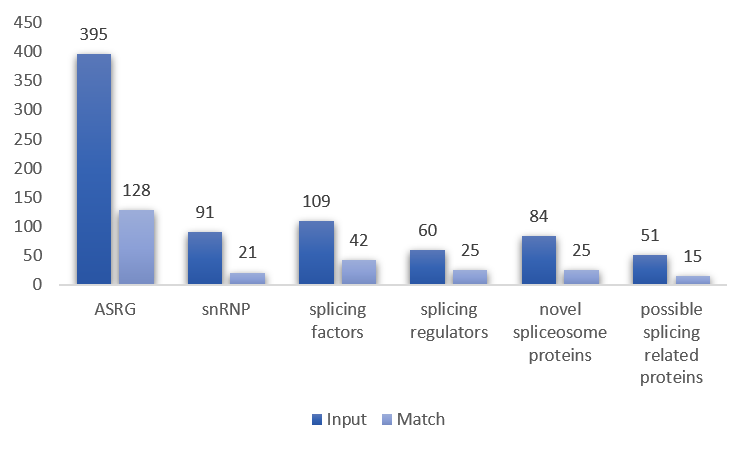

Supplement: Supplementary Figure 2 — Summary of sQTL cognate genes overlapping with splicing related genes. The x-axis shows the type of splicing related genes and Y-axis exhibits the number of input and matched splicing related genes. [file Image_2.tiff]

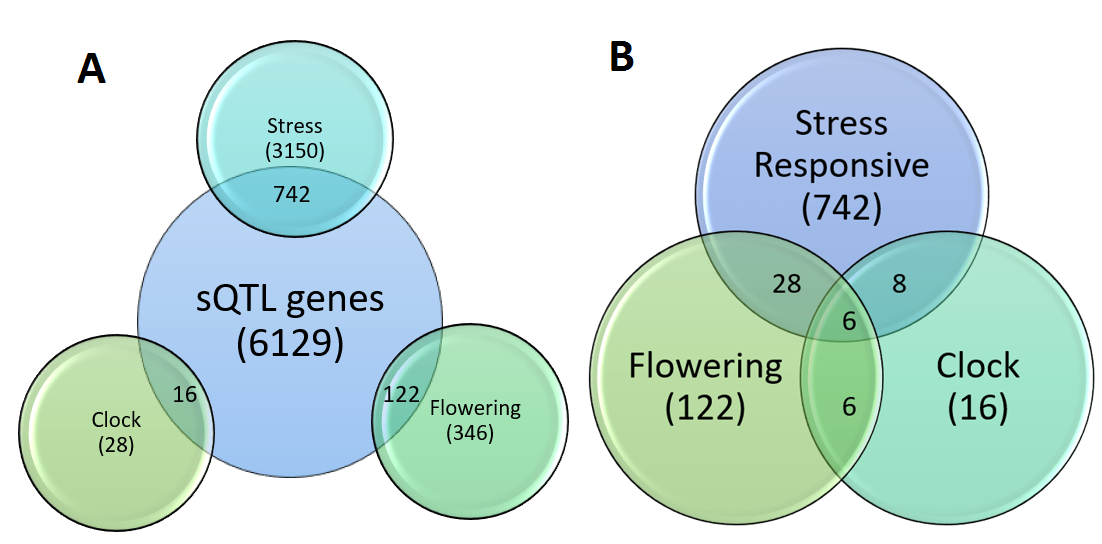

Supplement: Supplementary Figure 3 — Functional characterization of genes associated with sQTL. (A) sQTL cognate genes were highly enriched in different core functional categories (e.g. stress response, flowering). (B) Six genes were shared between different functional categories. [file Image_3.tiff]

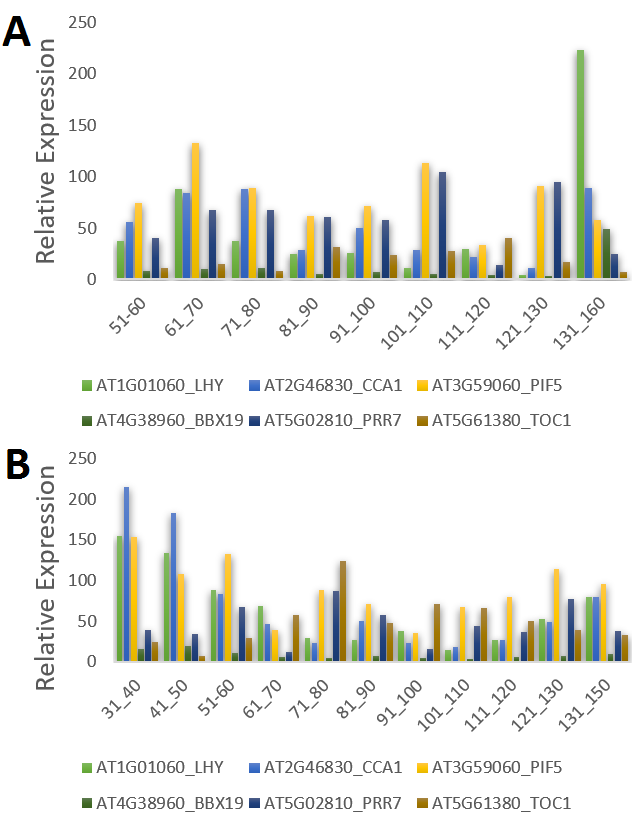

Supplement: Supplementary Figure 4 — Phenotypic association of six genes with flowering time. The x-axis shows days of flowering at 10 °C (A) and 16 °C (B) and Y-axis show the average/relative gene expression value for six genes across a diverse set of 666 A. thaliana accessions. [file Image_4.tiff]

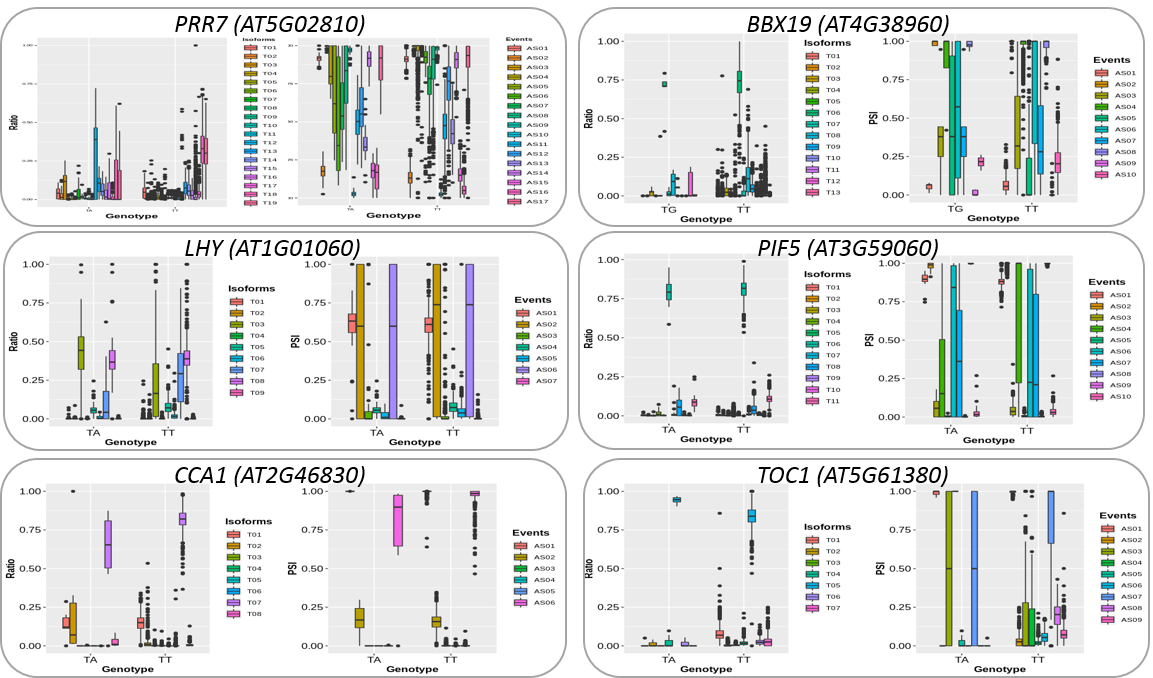

Supplement: Supplementary Figure 5 — The impact of sQTLs on splicing isoform ratios and AS events of six genes. The left panel shows the impact of change in genotype on splicing ratios of transcripts and the right panel shows the splicing events. For a detailed description of all isoforms and transcripts (see Supplementary Table 8 ). [file Image_5.tif]
