# Supplementary material for: Genome-Wide Identification of Splicing Quantitative Trait Loci (sQTLs) in Diverse Ecotypes of Arabidopsis thaliana
Source: Front Plant Sci. 2019 Oct 3;10:1160. doi: 10.3389/fpls.2019.01160 (PMC6785726; doi:10.3389/fpls.2019.01160)
Supplement: Supplementary Dataset 1 — RNA-Sequencing Analysis using genome as a reference [file DataSheet_1.docx]

**RNA-Sequencing Analysis using genome as a reference**

High quality filtered RNA-seq data was processed using complementarity approaches to generate genome and transcriptome-based expression datasets. The advancements in RNA-seq technology facilitated generation of a new reference transcriptome datasets and quantification methods for better RNA-seq analyses. Recently, for the accurate estimation of transcript abundance, a non-redundant reference transcript dataset for Arabidopsis AtRTD2 was built known as AtRTD2. It contains 82,910 transcript isoforms of 34,212 genes and a higher number (49%) of protein-coding genes as compared to the other reference datasets e.g. TAIR10 (21%) and Araport (41%). We used AtRTD2 as reference transcriptome and quantified transcriptome based expression of highly diverse accessions of *A. thaliana* using Salmon (Patro et al., 2017). The Salmon index was built using an ATRTD2 reference transcriptome. The indexed genome was then used to quantify transcriptomic expression of diverse ecotypes of *A. thaliana* using default parameters*.* The usage of **wicked-fast/memory efficient** transcript quantification tool and highly compact reference dataset provides a rapid way to generate an expression dataset but mapping against the transcriptome does not take novel events into account.

To resolve this problem, we also mapped filtered reads against the TAIR10 reference genome using STAR (Dobin et al., 2013). The STAR index was generated using the TAIR10 genome sequence and TAIR10 reference gene annotations. The 2-pass mapping mode was used to allow more splice junction reads to map to novel junctions with parameters “--runThreadN 8 --outFilterMultimapNmax 1 --readFilesCommand zcat --sjdbOverhang 99 --outSAMprimaryFlag AllBestScore --outFilterMismatchNmax 2/0 --outSJfilterCountTotalMin 10 5 5 5 --outSAMstrandField intronMotif --outFilterIntronMotifs RemoveNoncanonical --alignIntronMin 20 --alignIntronMax 12000 --outSAMtype BAM SortedByCoordinate --twopassMode Basic”. The transcriptome assembly for all accessions was performed using Stringtie and the assemblies were merged to generate a uniform set of assembled transcripts (**Supplementary Dataset 4)** (Pertea et al. 2015). The merged assembly was then used as a reference gene model to quantify the genome-based expression dataset for sQTL analysis.
